# A comparative dosimetric evaluation of two image-guided calibration strategies for multi-isocenter total body irradiation: integrated fan-beam CT–based alignment versus sequential cone-beam CT–guided localization

**DOI:** 10.3389/fonc.2026.1760909

**Published:** 2026-03-04

**Authors:** Bo Gao, Yaling Hong, Junjing Yan, Xiwei Chen, Suhua Tan, Yutao Zhu, Yan Liu, Zhaojie Yao, Lizhen Wu, Haiyan Chen, Zhanquan Lei

**Affiliations:** 1 Department of Radiation Oncology, Fujian Children’s Hospital (Fujian Branch of Shanghai Children’s Medical Center), College of Clinical Medicine for Obstetrics & Gynecology and Pediatrics, Fujian Medical University, Fuzhou, Fujian, China; 2 Department of Radiation Oncology, Shanghai Children’s Medical Center Affiliated to Shanghai Jiaotong University School of Medicine Institute, Shanghai, China; 3 Hunan Provincial Hospital of Integrated Traditional Chinese and Western Medicine, Changsha, Hunan, China

**Keywords:** children, cone-beam CT, dosimetry, fan-beam CT, image guidance, total body irradiation

## Abstract

**Background:**

Image-guided radiotherapy (IGRT) is key to making sure the radiation dose is accurate when treating kids with multi-isocenter total body irradiation (TBI). Ensuring dose accuracy in pediatric multi-isocenter TBI. Shanghai Children’s Medical Center employed low-dose fan-beam CT (FBCT) for integrated multi-isocenter calibration, whereas Fujian Children’s Hospital used cone-beam CT (CBCT) for sequential isocenter alignment.

**Objective:**

To evaluate FBCT versus CBCT guidance effects on target coverage and organ doses across two regimens: 12 Gy in 6 fractions and 3 Gy in 1 fraction.

**Methods:**

Retrospective analysis of 34 pediatric TBI patients (21 FBCT, 13 CBCT) treated with identical field setups (three upper-body isocenters; two/three lower-body isocenters; junction at upper femur third). Pre-treatment FBCT/CBCT images were registered to planning CTs; doses recalculated using original plans. Metrics: PTV V90%, V100%, V110%, mean doses; homogeneity index (HI); conformity index (CI); mean lung and kidney doses.

**Results:**

In the 12 Gy group, FBCT guidance improved PTV coverage: V_90%_ increased from 96.11% to 97.14%, V_100%_ from 90.40% to 92.81%, and V_110%_ decreased from 20.73% to 16.67% (all P < 0.01). HI decreased from 0.25 to 0.16, CI increased from 0.77 to 0.89, and mean PTV dose rose from 12.33 to 12.57 Gy (all P < 0.01). Mean lung dose fell from 8.61 to 8.47 Gy, and mean kidney dose from 8.24 to 8.10 Gy (both P < 0.01). In the 3 Gy group, FBCT guidance also improved PTV coverage: V_90%_ increased from 96.32% to 97.82%, V_100%_ from 91.44% to 93.97%, and V_110%_ decreased from 17.43% to 13.72% (all P < 0.01). HI decreased from 0.21 to 0.13, CI increased from 0.77 to 0.87, and mean PTV dose rose from 3.08 to 3.12 Gy (all P < 0.01). Mean lung dose decreased from 2.34 to 2.25 Gy, and mean kidney dose from 2.09 to 2.06 Gy (both P < 0.01).

**Conclusion:**

FBCT guidance gave better target dose conformity and homogeneity, and lower lung doses, than CBCT guidance—both for the 12 Gy myeloablative regimen and the 3 Gy low-dose regimen. These results suggest that FBCT guidance is a better option for image-guided total body irradiation in children.

## 1 Introduction

Total body irradiation (TBI) is a standard part of the treatment before hematopoietic stem cell transplantation [cf (1).]. It is commonly used for children with acute leukemia or aplastic anemia. TBI works by killing remaining cancer or abnormal blood cells and weakening the patient’s immune system to help the new stem cells take hold [cf (2).]. In traditional TBI (also called 2D-TBI), large radiation fields are used—front-to-back or side-to-side—at a long distance from the machine to the skin (usually over 2 meters). Lead blocks are placed in the beam to protect the lungs and kidneys, lowering the risk of lung or kidney damage [cf (3).]. Newer methods like helical tomotherapy (HT) [cf (4, 5).], volumetric modulated arc therapy (VMAT) [cf (6, 7).], and fixed-angle intensity-modulated radiotherapy (IMRT) can also deliver TBI [cf (8).]. IMRT shapes the radiation dose more precisely, reducing dose to sensitive organs like the lungs and kidneys while still giving full dose to the target. This helps lower side effects without reducing treatment effectiveness—and may improve long-term outcomes [cf (8, 9).].

Linear accelerators have a limited radiation field size. To deliver TBI with VMAT, multiple isocenters are needed—usually three for the upper body (head, chest, and abdomen) and two or three for the lower body, with the junction at the top of the femur [cf (6, 7).]. This method covers the whole body but makes it hard to match doses smoothly at the junctions. Even small setup errors can lead to areas in the target that get too little or too much radiation [cf (10).].

Image-guided radiotherapy (IGRT) is now standard for TBI to correct daily positioning differences. Most centers use kilovoltage cone-beam CT (CBCT)[cf (11).]; low-dose fan-beam CT (FBCT) is a newer option [cf (12).]. With CBCT, you need a separate scan and alignment for each isocenter. That takes more time and makes it hard to keep all isocenters lined up correctly relative to each other. FBCT, on the other hand, takes one low-dose scan that covers the full length of the body—just like the plan CT (pCT) [cf (12).]. One alignment to the original pCT then tells the system how to move the treatment couch to line up all isocenters at once. This gives real whole-body correction.

Both FBCT and CBCT are used in children receiving TBI, but no study has directly compared how they affect radiation dose—especially to the target, lungs, and kidneys. This study compares two real-world approaches: the FBCT method used at Shanghai Children’s Medical Center, which aligns all isocenters in one step, and the CBCT method used at Fujian Children’s Hospital, which adjusts each isocenter separately. We test both methods for two common TBI schedules: 12 Gy in fractions (myeloablative) and 3 Gy in 1 fractions. The main outcomes are how much of the target receives at least 95% of the prescribed dose, and the average dose to both lungs and both kidneys. Results will help teams choose the best image-guidance method for children having TBI.

## 2 Patients and methods

### 2.1 Patient information

We included 34 children who received total body irradiation (TBI) at Shanghai Children’s Medical Center or Fujian Children’s Hospital between January 2023 and August 2025. The children were divided into two groups: 18 received 12 Gy in 6 fractions (12 from Shanghai and 6 from Fujian), and 16 received 3 Gy in 1 fraction (9 from Shanghai and 7 from Fujian). Inclusion criteria were age 3 to 18 years, diagnosis of ALL, AML, AA, or other conditions requiring TBI before transplant, completion of the full treatment course, and availability of complete image-guided radiation therapy (IGRT) images and dose data. We excluded children with major setup errors, poor image quality, or who withdrew from treatment. **Table 1** shows the two groups did not differ significantly in sex, age, radiotherapy technique, diagnosis, or other factors (all P > 0.05).

**Table 1:** Baseline characteristics of the patients V_90%_ represents the percentage of the total target volume in the area where more than 90% of the prescribed dose is administered, and V_100%_, V_110%_ similarly, Dmax refers to the highest dose in the target.

Characteristic		FBCT group n = 21	CBCT group n = 3	P
Age (years)		12.8	12.4	0.72
Male(n, %)		12 (57.1%)	8 (61.5%)	0.13
Technology		VMAT	VMAT	—
Diagnosis: ALL/AML		12	6	—
Diagnosis: SAA		9	7	—
Mean V_90%_(%)	12Gy	98.15	98.12	0.49
		98.44	98.43	0.44
Mean V_100%_(%)	6Gy	93.34	93.20	0.37
		94.68	94.77	0.32
Mean V_110%_(%)	12Gy	7.37	7.25	0.37
		5.82	5.70	0.25
PTV mean dose	6Gy	12.55	12.52	0.69
		3.09	3.08	0.75
Lungs mean dose	12Gy	7.89	7.91	0.28
		2.05	2.09	0.35
Kidneys mean dose	6Gy	7.68	7.69	0.31
		1.95	1.96	0.40

### 2.2 Radiotherapy planning design method (consistent in two centers)

**Figure 1:**
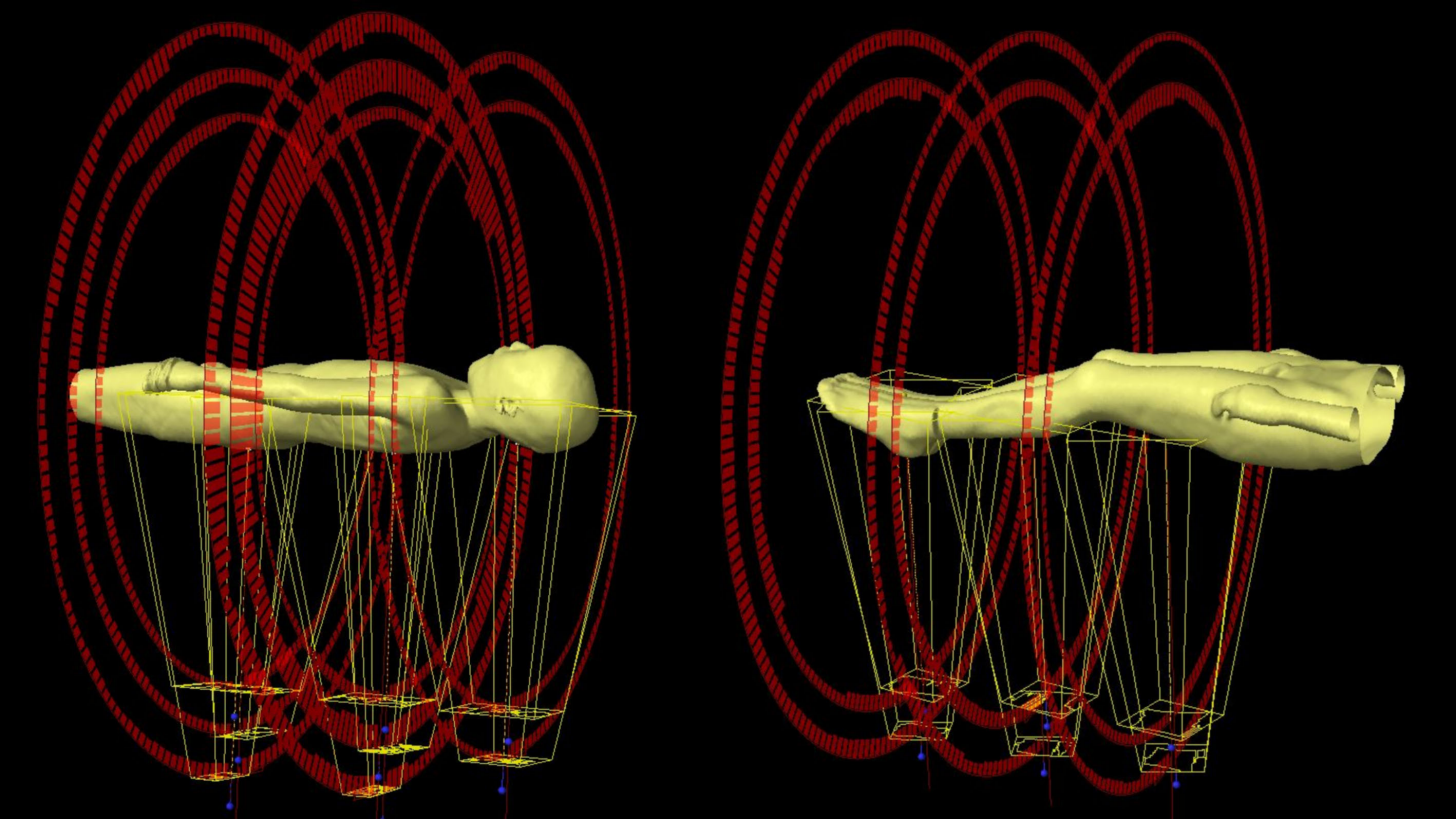
Schematic diagram of the field arrangement for radiotherapy planning.

**Figure 2:**
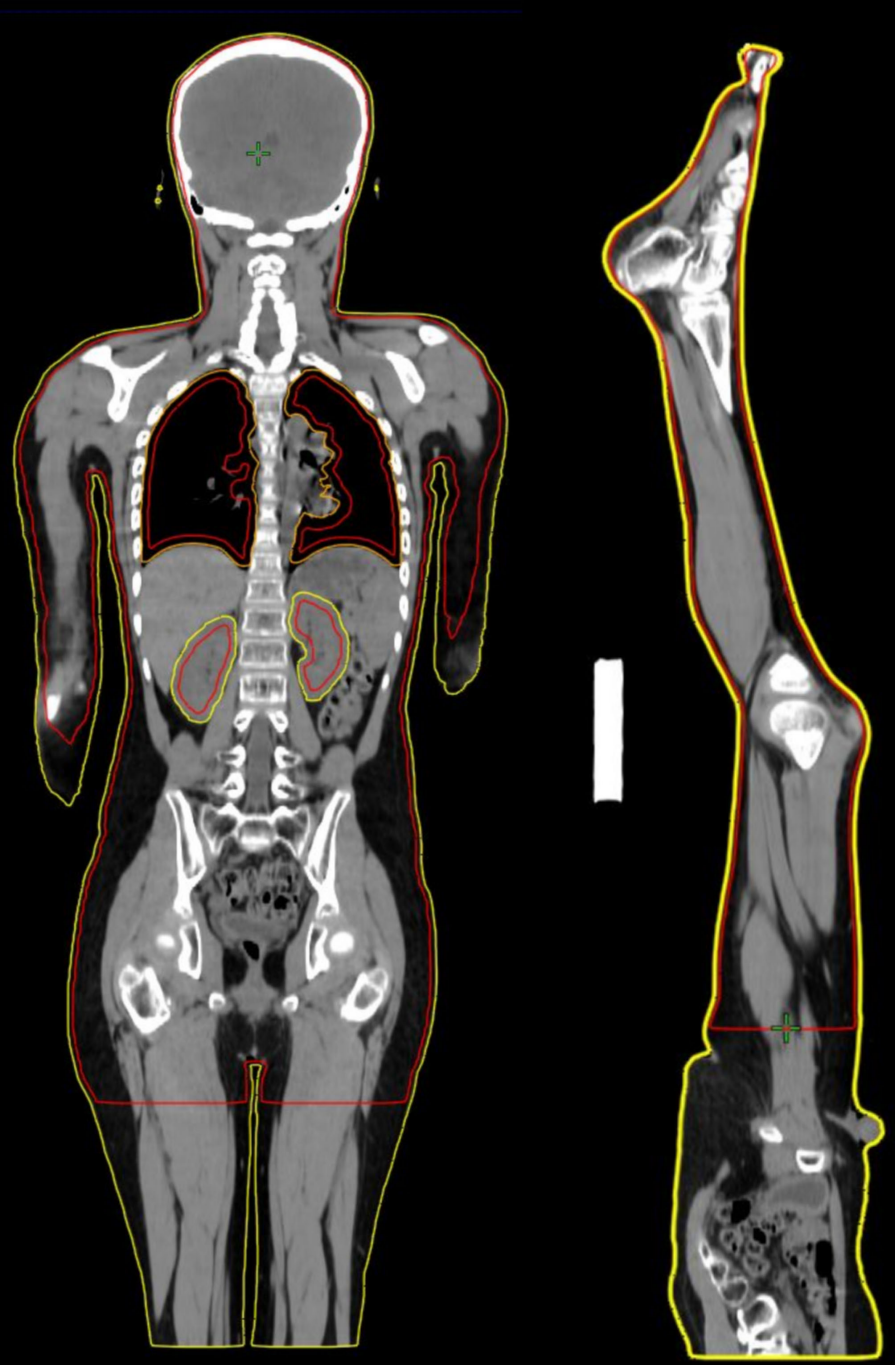
TBI target area. The red line segment in the figure encloses the PTV of the TBI target area, and the outermost yellow line represents the body surface contour. The outer contour of the body surface is indented by 3mm, and the lens and the area 3mm outside the outer contour of the lens are removed, as well as the area 5mm inside the outer contour of the lungs and the area 5mm inside the outer contour of the kidneys. The resulting area is the PTV of the TBI target area.

Both centers are partner hospitals in a shared clinical research program. All TBI plans were created and reviewed by the same team of medical physicists and radiation oncologists, ensuring consistent planning and quality control across sites. Shanghai Children’s Medical Center used uTPS (United Imaging Healthcare, version 1520); Fujian Children’s Hospital used Varian Eclipse (Siemens Healthineers, version 15.5), as shown in **Figure 1**. For VMAT delivery, the upper body used three isocenters (two full 360° arcs for the head, three for the chest, and three for the abdomen), and the lower body used two to three isocenters (two arcs each for the thighs, knees, and calves). The field junction was placed at the upper third of the femur. Target volumes followed standard clinical contouring guidelines and are illustrated in **Figure 2**. Plan acceptance criteria were: PTV V90% > 95%, PTV V100% > 90%, and PTV Dmax < 120% of the prescription dose. Here, V90% and V100% refer to the percentage of PTV volume receiving at least 90% or 100% of the prescription dose. Organ-at-risk limits were: mean lung dose < 66.7%, mean kidney dose < 83.3%, and lens dose < 50% of the prescription dose. The maximum dose rate was 100 MU/min; the average dose rate to the lungs was about 10 cGy/min. While some studies suggest that lower dose rates may reduce the risk of radiation pneumonitis [cf (13).], none of the 47 patients developed acute radiation pneumonitis. This observation was not part of the study’s primary objectives and is not discussed further.

As shown in **Figure 2**, the area surrounded by the red line is the TBI target area (irradiation range), namely the PTV, which is defined as:


$$\text{PTV}={\text{Body}}_{\text{i}\_3\text{mm}}-{\text{Lungs}}_{\text{i}\_5\text{mm}}-{\text{Kidneys}}_{\text{i}\_5\text{mm}}-{\text{Lens}}_{\text{o}\_3\text{mm}}$$

Here, Body_i_3mm_ represents the area with a 3mm inward contraction from the outer contour of the body surface, Lungs_i_5mm_ represents the area with a 5mm inward contraction from the outer contour of both lungs, Kidneys_i_5mm_ represents the area with a 5mm inward contraction from the outer contour of both kidneys, and Lens_o_3mm_ represents the area with a 3mm outward expansion from the outer contour of the lens.

### 2.3 TBI treatment and IGRT methods

The patient was immobilized using a thermoplastic head mask and a vacuum cushion, with auxiliary skin markings placed on the body to facilitate reproducible setup. Treatment was delivered in two distinct positional configurations: head-first supine (HFS) for the upper-body target volume, followed by feet-first supine (FFS) for the lower-body target volume. Prior to each treatment fraction, volumetric image guidance was performed. All image registrations were independently reviewed and approved by a board-certified radiation oncologist; treatment proceeded only after formal sign-off. The institutional image-guidance protocols are summarized below:

Shanghai Children’s Medical Center: Integrated multi-isocenter FBCT calibration. Equipment: uRT-linac 506c (United Imaging Healthcare) — A device integrating FBCT and a linear accelerator, 1-meter longitudinal FBCT volumes in a single continuous scan.

Workflow: (1) A single low-dose FBCT scan covering the entire treatment region is acquired immediately before treatment; (2) the FBCT is rigidly registered to the pCT as a unified volume; (3) the system computes translational and rotational offsets for all planned isocenters simultaneously, preserving their relative spatial relationships; (4) the couch is adjusted once—based on the composite correction vector—to align all isocenters globally; (5) no additional per-isocenter verification scans are performed.

Fujian Children’s Hospital: Sequential single-isocenter CBCT calibration. Equipment: Varian TrueBeam (Varian Medical Systems, now part of Siemens Healthineers) — equipped with an on-board CBCT.

Workflow: (1) A localized CBCT scan is acquired at each individual isocenter location; (2) each CBCT is rigidly registered to its corresponding anatomical region in the pCT; (3) the couch is repositioned separately for each isocenter based on its unique registration result; (4) inter-isocenter geometric consistency is not enforced during registration or couch movement—each is treated as an independent setup.

### 2.4 Dose recalculation and evaluation

The patients at Shanghai Children’s Medical Center used their current IGRT images to locate the beam isocenter on the FBCT. Subsequently, the beam was transferred to the FBCT for dose recalculation to estimate the actual radiation dose distribution received by the patient at this treatment stage. At Fujian Children’s Hospital, the Varian Velocity software (version 4.1) was used to generate a registered CT (rCT) through deformable registration. First, the pCT and CBCT were rigidly aligned. Then, Velocity performed deformable registration between them to compute a deformation vector field. Using this field, the PTV and organs at risk were mapped from the pCT onto the CBCT. A senior radiation oncologist reviewed and confirmed the accuracy of these contours on the CBCT. Next, the inverse of the deformation field was applied to the pCT to produce the rCT. Finally, dose was recalculated on the rCT to estimate the actual dose distribution for that session.

Evaluation metrics included: PTV: V_90%_ (percentage of PTV receiving ≥90% of the prescribed dose), V_100%_, V_110%_, PTV mean dose, homogeneity index HI (per ICRU Report 83; lower values indicate better uniformity), and conformity index CI (per Paddick definition; values closer to 1 indicate better conformity). Organs at risk: mean lung dose and mean kidney dose. Statistical analysis was done using SPSS 26.0. Continuous variables are reported as mean ± standard deviation. Group comparisons used the Wilcoxon rank sum test. A p-value < 0.05 was considered statistically significant.

## 3 Results

As shown in **Table 1**, the two groups were well balanced with respect to age (12.8 vs 12.4 years), sex distribution (57.1% vs 61.5% male), diagnosis (ALL/AML: 12 vs 6; AA: 9 vs 7), radiotherapy technique, and prescribed dose (P > 0.05). No significant differences were found in PTV dose metrics (V_90%_, V_100%_, V_110%_, mean dose) or mean doses to both lungs and both kidneys.

As shown in **Table 2**, in the 12 Gy/6F regimen, the FBCT-guided group achieved superior target coverage and organ sparing compared with the CBCT-guided group: V_90%_ was 97.14% vs 96.11% (P < 0.01); V_100%_ was 92.81% vs 90.40% (P < 0.01); HI was 0.16 vs 0.25; CI was 0.89 vs 0.77. Mean lung dose was reduced by 0.14 Gy (8.47 vs 8.61 Gy), and mean kidney dose was reduced by 0.14 Gy (8.10 vs 8.24 Gy); all differences were statistically significant (P < 0.05).

**Table 2:** Dosimetric results for the 12 Gy in 6 fractions regimen.

	PTV dose						Lungs mean dose (Gy)	Kidneys mean dose (Gy)
Parameter	V90% (%)	V100% (%)	V110% (%)	mean dose(Gy)	HI	CI		
FBCT group	97.14 ± 0.50	92.81 ± 1.15	16.67 ± 2.87	12.57 ± 0.25	0.16 ± 0.03	0.89 ± 0.03	8.47 ± 0.28	8.10 ± 0.22
CBCT group	96.11 ± 1.20	90.40 ± 3.80	20.73 ± 4.21	12.33 ± 0.44	0.25 ± 0.03	0.77 ± 0.05	8.61 ± 0.34	8.24 ± 0.26
P	<0.01	<0.01	<0.01	<0.01	<0.01	<0.01	<0.01	<0.01

As shown in **Table 3**, the 3 Gy/1F regimen showed consistent improvements: V_100%_ was 93.97% vs 91.44% (P < 0.05); HI was 0.13 vs 0.21; CI was 0.87 vs 0.77; mean lung dose was 2.25 vs 2.34 Gy (P < 0.05); mean kidney dose was 2.09 vs 2.11 Gy (P < 0.05). Importantly, V_110%_ was lower in the FBCT group for both regimens, indicating a more homogeneous dose distribution and reduced risk of local overdosing. All reported dosimetric differences were statistically significant (P < 0.05).

**Table 3:** Dosimetric results for the 3 Gy in 1 fractions regimen.

	PTV dose						Lungs mean dose (Gy)	Kidneys mean dose (Gy)
Parameter	V90% (%)	V100% (%)	V110% (%)	mean dose(Gy)	HI	CI		
FBCT group	97.82 ± 0.44	93.97 ± 0.93	13.72 ± 1.96	3.12 ± 0.11	0.13 ± 0.02	0.87 ± 0.04	2.25 ± 0.12	2.09 ± 0.06
CBCT group	96.32 ± 2.63	91.44 ± 3.10	17.43 ± 3.50	3.08 ± 0.12	0.21 ± 0.03	0.77 ± 0.06	2.34 ± 0.15	2.11 ± 0.05
P	<0.01	<0.01	<0.01	<0.01	<0.01	<0.01	<0.01	<0.01

## 4 Discussion

This is the first study to directly compare FBCT-based multi-center integrated image guidance with CBCT-based center-specific calibration for pediatric total body irradiation (TBI), using both the myeloablative (12 Gy/6F) and low-dose (3 Gy/1F) regimens. FBCT guidance consistently improved target coverage, dose homogeneity, and lung protection — regardless of the regimen used. The average difference between measured and planned doses was under 2% for both guidance methods (12 Gy group: Fujian protocol — 12.57 Gy vs. 12.52 Gy). In contrast, Patel et al. [cf (14).] reported a ~4% discrepancy, Kawa-Iwanicka et al. [cf (15).] reported >7%, and Sarkar et al. [cf (16).] found a 10.4% deviation when using extended SSD for TBI. These findings confirm that both CBCT and FBCT guidance achieve clinically acceptable dose accuracy in pediatric TBI.

The main challenge in TBI is maintaining uniform dose across the entire body. Linear accelerator fields are typically under 40 cm, so full-body coverage requires multiple isocenters. Bo Gao et al. [cf (10).] and Qilin Li et al. [cf (17).] reported cold spots and hot spots at beam junctions due to setup errors. CBCT-based IGRT registers only one isocenter at a time. It does not account for how other isocenters move relative to it. For example, if the chest isocenter moves 1 mm forward and the abdomen isocenter moves 1 mm backward, the junction shifts by 2 mm. This causes dose gradients to deviate from the plan, reducing V_90%_ and increasing V_110%_. Yongqiang Zhou et al. [cf (18).] also found underdosing and overdosing at junctions in multi-isocenter treatments. FBCT (e.g., United Imaging uCT-ART) captures a single low-dose image up to 1 m long [cf (19).], this image matches the pCT geometry. The system uses this match to compute offsets for all isocenters at once. Then it moves the treatment couch as one unit. This prevents misalignment between isocenters.

The mean lung dose is an independent predictor of radiation pneumonitis. Wong et al. [cf (20).] found that controlling the mean lung dose below 8–10 Gy significantly reduces the incidence of radiation pneumonitis. Guo et al. [cf (21).] showed that intensity-modulated radiotherapy (IMRT) techniques such as VMAT-TBI reduce the mean lung dose from 10.8 ± 0.7 Gy to 9.4 ± 0.8 Gy. In this study, lung doses estimated using IGRT imaging were 8.61 Gy for the CBCT group and 8.47 Gy for the FBCT group, a reduction of 0.14 Gy. Lower lung doses likely offer better protection for the lungs. Even with a 3 Gy single-fraction regimen, the FBCT group had a mean lung dose 0.09 Gy lower than the CBCT group, suggesting FBCT may provide long-term benefits by reducing late lung function damage.For renal tissue, Kal et al. [cf (22).] proposed a biological effective dose (BED) tolerance threshold of approximately 16 Gy, equivalent to a mean dose limit of 10.56 Gy for the 12 Gy/6F fractionation schedule. In this study, the maximum mean renal dose was 8.61 ± 0.34 Gy in the 12 Gy/6F CBCT group, which remains below the reported limit. Shahid et al. [cf (23).] reported mean left and right renal doses of 9.17 Gy and 8.05 Gy when using VMAT for total bone marrow irradiation, values comparable to those in this study. FBCT also showed a dose advantage for the kidneys. Beyond dose benefits, FBCT has clear advantages in operational efficiency and imaging dose. A single FBCT scan takes less than 60 seconds to complete. This is clinically important for pediatric patients, especially those requiring sedation or with difficulty cooperating, because it shortens treatment time and reduces additional radiation exposure.

This study is a retrospective analysis with a limited sample size, and dynamic factors such as respiratory motion were not included. Additionally, the study primarily evaluated dosimetric differences between FBCT- and CBCT-guided total body irradiation, demonstrating the dose advantages of FBCT’s integrated long-target imaging guidance; however, it did not propose solutions to mitigate these dosimetric differences. Finally, the clinical adoption of FBCT remains constrained by limited equipment availability, as the technique is currently implemented in only a few radiotherapy centers worldwide.

## 5 Conclusion

FBCT-enabled multi-isocenter integrated image guidance demonstrates clear advantages over traditional sequential isocenter setup with CBCT in pediatric total body irradiation (TBI). This approach enhances target dose coverage and uniformity across prescription regimens while reducing mean lung dose, representing a promising strategy for precise and safe TBI delivery.

## Data Availability

The raw data supporting the conclusions of this article will be made available by the authors, without undue reservation.
